# Assessment of the appropriateness of the i-CONSENT guidelines recommendations for improving understanding of the informed consent process in clinical studies

**DOI:** 10.1186/s12910-021-00708-1

**Published:** 2021-10-13

**Authors:** Jaime Fons-Martinez, Cristina Ferrer-Albero, Javier Diez-Domingo

**Affiliations:** 1 Vaccine Research Area, Foundation for the Promotion of Health and Biomedical Research of Valencia Region, FISABIO, Avda. de Catalunya, 21, 46020 Valencia, Spain; 2 Facultad de Medicina y Ciencias de la Salud, Universidad Católica de Valencia San Vicente Mártir, Valencia, Spain

**Keywords:** Informed consent process, i-CONSENT, RAND/UCLA method, Ethical guidelines, Ethical recommendations

## Abstract

**Background:**

The H2020 i-CONSENT project has developed a set of guidelines that offer ethical recommendations and practical tools aimed at making the informed consent process in clinical studies more comprehensive, tailored, and inclusive. An analysis of the appropriateness of some of its novel recommendations was carried out by a group of experts representing different stakeholders.

**Methods:**

An adaptation of the RAND/UCLA Appropriateness Method was used to assess the level of agreement on the recommendations among 14 representatives of different stakeholders, including patients, regulators, investigators, ethics experts, and the pharmaceutical industry. The process included two rounds of rating and a virtual meeting.

**Results:**

Fifty-three recommendations were evaluated. After the first round, 34 recommendations were judged “appropriate”; 19 were judged “uncertain”; and none was judged “inappropriate”. After the second round, 9 “uncertains” changed to “appropriate”. All recommendations rated medians of 6.5–9 on a 1–9 scale (1 = “extremely inappropriate”, 5 = “uncertain”, 9 = “extremely appropriate”). The sections “General recommendations” and “Gender perspective during the consent process for clinical studies” showed the highest “uncertainty” rating. The four keys to improving the understanding of the ICP in clinical studies are to: (1) consider consent a two-way continuous interaction that begins at the first contact with the potential participant and continues until the end of the study; (2) improve investigators’ communication skills; (3) co-create the information; and (4) use a layered approach, including information to compensate for the potential participant’s possible lack of health literacy and a glossary of terms.

**Conclusions:**

The RAND/UCLA method has demonstrated validity for assessing the appropriateness of recommendations in ethical guidelines. The recommendations of the i-CONSENT guidelines were mostly judged “appropriate” by all stakeholders involved in the informed consent process.

**Supplementary Information:**

The online version contains supplementary material available at 10.1186/s12910-021-00708-1.

## Background

The informed consent process (ICP) is one of the most important contributions of ethics in the field of clinical research. It ensures the autonomy of potential participants in their decision to participate in a study or to withdraw at any time without consequences.

The Belmont Report [1] identified three main ICP components: information, comprehension, and voluntariness. Fulfilling all these components is challenging. Information is a key element, in terms not only of its content but also its presentation. Proper understanding of this information must be ensured, so that an individual can make an informed decision, and the ICP must necessarily be free of coercion and undue influence, in order to ensure voluntariness.

Patient information and consent forms are increasingly long and difficult to understand. They are usually written in complex language (above the recommended grade 8 reading level) and often omit significant information [2, 3].Several studies have reported a lack of understanding of some content [4, 5], and no significant advances have been made in recent decades [6].

Despite the fact that informed consent, in addition to its informative purpose, is nowadays also used as the document that legally regulates the relationship between all parties involved in the study, some aspects must still be improved to ensure clear communication between participants and investigators. Proper information and efficient communication are mainstays for upholding the fundamental ethical principle of respect for the participant’s autonomy.

Several guidelines and legal documents have been published on the consent process, addressing what informed consent is and should be, why is it important in clinical studies, the main procedures to follow during the ICP, and the minimum content to be covered. In accordance with these documents, the H2020 project i-CONSENT has developed a set of guidelines that provide ethical recommendations and practical tools that aim to make the ICP in clinical studies more comprehensive, tailored, and inclusive. The “Guidelines for Tailoring the Informed Consent Process in Clinical Studies” [7] (i-CONSENT guidelines) have been prepared from a review of the literature and based on the opinion of various experts (more information about the elaboration of the guidelines and the project is available on the project website [8] and in CORDIS [9]).

During the project, multiple literature reviews and systematic reviews were conducted to identify methods and strategies to improve informed consent, including the use of new technologies. Aspects of informed consent related to age and gender were also investigated, as well as socio-cultural perspectives on the notion of autonomy and other fundamental principles of informed consent. Ethical and legal issues related to the informed consent process were explored, including the review of the main international guidelines for medical research and the legal framework at national level of 6 countries (Austria, Spain, Italy, France, Germany, the UK) and the EU, in particular for women and minors involved in clinical research.

In addition, through different workshops and patient centered techniques the opinion of experts and representatives of the different stakeholders about different aspects of the informed consent process has been gathered. This information has allowed filling some of the gaps found in the literature and getting the perspectives of the main stakeholders about different aspects of the informed consent process.

All this input has been used in the development of the *“*Guidelines for Tailoring the Informed Consent Process in Clinical Studies*”.* Most novel recommendations were extracted from these guidelines, and their appropriateness was analysed by a group of representatives from different stakeholders using an adaptation of the RAND/UCLA methodology. This study was performed to increase the quality of the recommendations included in the guidelines and made a very important contribution to the final guidelines. The validation of the guidelines by experts representing different stakeholders has been considered a key step prior to the final drafting of the guidelines.

## Methods

An adaptation of the RAND/UCLA Appropriateness Method [10], identified by several authors as the best consensus method for developing guidelines and recommendations [11], was used to assess the level of agreement of representatives from different stakeholders on the recommendations for improving the understanding of the ICP in clinical studies, extracted from the i-CONSENT guidelines.

The expert panel comprised 14 representatives from different stakeholders, including patients, regulators, investigators, ethics experts, and the pharmaceutical industry.

Participants were selected according to their experience in relevant institutions or their prominence in the scientific literature. They were asked to give their own view, not that of their institutions.

The criteria follow to choose the participants were:

- Investigators: A review of authors from European organisations with articles in the field of informed consent was carried out, the authors considered most suitable in view of their published articles were selected and contacted by email.
- Patients: The European Patients' Academy on Therapeutic Innovation (EUPATI) was contacted and asked to forward information to their fellows and trainees so that those interested in participating could contact us. Several applications were received and those whose profiles were considered most interesting were chosen, including aspects such as membership of patient associations, chronic patient status or being a patient representative for other bodies (including regulatory bodies).
- Regulators: Representatives of national and international medicines agencies were contacted. Given the global pandemic situation, it was very difficult to get positive responses. Finally, the participation of a person from the EMA with a profile of interest to the study was secured.
- Ethics experts: Members of reputable ethics networks and bodies were selected. Two of the 3 experts included have an extensive scientific output on informed consent and research ethics; the third has a profile closer to regulation, which was considered optimal given the difficulties in contacting regulators.
- Pharmaceutical industry: Informed consent experts were selected from the pharmaceutical industry, including experts in this field from the Transcelerate Biopharma initiative, through GSK and EFPIA members.

A set of 30 recommendations, 53 including the sub-recommendations, were divided into 10 sections, including the 5 ICP steps specified in the i-CONSENT guidelines, as follows:

- General recommendations
- Recommendations for the preparation of information
- Step 1: First contact with the potential participant
- Step 2: Provision of information
- Step 3: Discussion and decision-making
- Step 4: Intervention and follow-up
- Step 5: End of the study
- The gender perspective during the consent process for clinical studies
- ICP in clinical studies involving minors
- ICP in clinical studies involving people from different cultural and religious backgrounds

**Fig. 1:**
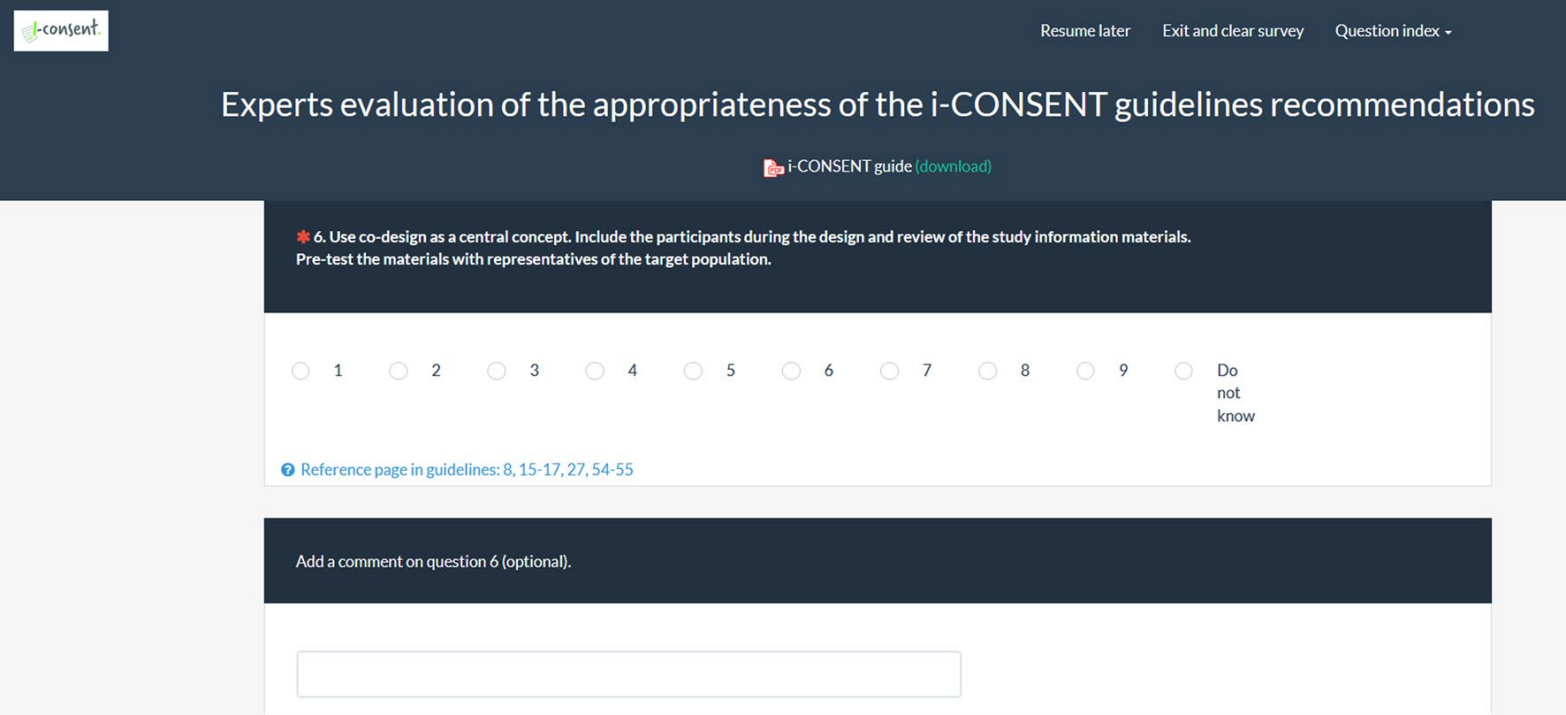
Screenshot of the platform used to complete the survey

The experts were asked to rate the appropriateness of each recommendation from 1 to 9, where 1 is "extremely inappropriate" and 9 is "extremely appropriate" (appropriateness scale: 1 = “extremely inappropriate”, 5 = “uncertain”, 9 = “extremely appropriate”). A “Do not know” option was added, for use only when the question was outside the respondent’s field of expertise. Ratings were made with an average potential participant and an average clinical study in mind, focusing on the recommendation's effectiveness, without considering cost implications. The survey was completed on an electronic platform (Fig. 1).

**Fig. 2:**
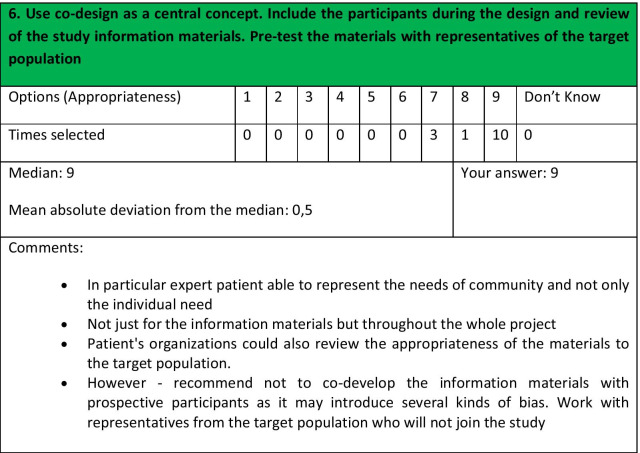
Example of the information about a recommendation in the PPRS

The process included two rounds of rating, as follows:

1. Fourteen experts agreed to participate after receiving a detailed explanation of the RAND process.
2. First round of rating: panellist received the link and instructions on how to complete the survey. They were given 3 weeks to complete the survey and submit their responses.
3. A Personalised Panellist Rating Sheet (PPRS) was prepared and sent to each panellist. It included the frequency of responses for each recommendation, the median, the mean absolute deviation from the median, their own response, and the comments included by the panellists on each recommendation (see example in Fig. 2).
4. A virtual meeting of the panel of experts with a second round of rating was held on an online platform. The aim was to discuss the recommendations that had not achieved clear agreement after the first round of rating. The aim of the virtual meeting was not to force the panel to reach consensus and this was indicated to the panellists. Therefore, the aim was, on the one hand, for the panellists to be able to state their positions and express their doubts or suggestions; on the other hand, for the i-CONSENT team to clarify the reason and meaning of each recommendation, thus facilitated the correct understanding of the recommendations and ensured that all panellists evaluated the same thing. The virtual meeting allowed the different points of view to be presented, clarified and discussed. After discussing the recommendations, a second round of rating took place. Each panellist had access to their answers from the first round for this second round.

Levels of appropriateness and agreement were defined on the basis of the recommendations included in “The RAND/UCLA Appropriateness Method User's Manual” [10].

Appropriateness levels were determined by the median of the panel and the presence (or absence) of agreement. The original definition was modified to a more restrictive position, taking into consideration a lack of agreement (rather than the existence of disagreement) sufficient to consider a recommendation as “uncertain”. Median ratings falling exactly between the 3-point boundaries (3.5 and 6.5) were included in the higher appropriateness category.

Levels of appropriateness:

- “Appropriate”: panel median of 6.5–9, with agreement
- “Uncertain”: panel median of 3.5–6 OR any median without agreement
- “Inappropriate”: panel median of 1–3, with agreement

The definition of agreement or disagreement depended on the panel size and the distribution of the panellist ratings on the 3-point regions (Table 1). Because a “Don’t know” category of response was included, the panel size was calculated for each recommendation including only responses with a rating of 1–9.

**Table 1:** Definition of agreement and disagreement among panellists for different panel sizes

Panel size	Disagreement	Agreement
	Number of panellists rating at each extreme (1–3 and 7–9)	Number of panellists rating outside the 3-point region containing the median (1–3; 4–6; 7–9)
From 8 to 10	3 or more	2 or less
From 11 to13	4 or more	3 or less
From 14 to 16	5 or more	4 or less

*Source* “The RAND/UCLA Appropriateness Method User's Manual” [10]

## Results

All 14 panellists (10 women, 4 men) from 12 different countries (10 European, 2 non-European) representing 5 stakeholders^1^ (5 patient representatives; 1 regulator; 3 investigators; 3 ethics experts; 2 pharmaceutical industry representatives) submitted the survey on time during the 2 rounds of rating and all panellists attended the virtual meeting.

After the first round, 34 recommendations were considered “appropriate”; 19 were considered “uncertain”; 0 recommendations were considered “inappropriate”. The median of 52 recommendations was in the “appropriate” range and 1 was in the “uncertain” range.

The 19 recommendations with an “uncertain” level of appropriateness were discussed in the virtual meeting. The recommendations discussed were:

### General Recommendations

**Recommendation (Rec.) 2.** Feedback from participants:

- **Rec. 2.2**. Feedback should be obtained at all stages:
  - On the experience before starting the study (obtained during the first month of participation).
  - On the experience during the study (obtained during the trial).
  - On the experience at the end of the study (obtained at the last visit).
- **Rec. 2.3**. Conduct a debriefing session with your team about the consent process using this information:
  - A session held after the study may help to improve the consent process in future studies.
  - A session held during the study may also help to improve the process of the current study.
- **Rec. 4.** Digital and health literacy:
  - **Rec. 4.1**. Train your participants to improve their digital and health literacy.
  - **Rec. 4.3**. Use links to “further information”.
  - **Rec. 4.4.** Provide participants with information on how to detect fake news and unreliable sources.

### Recommendations for preparing information

- **Rec. 5.** Use interdisciplinary quantitative and qualitative methodologies to define your study population, interests, and needs. It may be useful to:
  - review the available literature on the target population (e.g., systematic or narrative literature review);
  - ask the target population directly (e.g. interviews, surveys, Design Thinking);
  - seek advice from experts (key informant interviews, brainstorming, etc.);
  - observe the target population; and/or
  - analyse their interactions on social media and blogs.
- **Rec. 11.** Provide references to reliable sources of information.
- **Rec. 12.** If using placebo, include a short description of the placebo effect (positive and negative).

### Step 1: First contact with the potential participant

- **Rec. 13**. Due to the growing use of digital technology among the population and the appearance of decentralised clinical trials, consider:
  - **Rec. 13.1**. Use of different channels to advertise the study:
    -
      - Social media
      - Email

### Step 3: Discussion and decision-making

- **Rec. 18**. Check that potential participants have understood all study information by:
  - Interview: Teach-back or teach-to-goal methods can be helpful.
  - Questionnaires, such as the Quality of Informed Consent (QuIC), Deaconess Informed Consent Comprehension Test (DICCT), or the Brief Informed Consent Evaluation Protocol (BICEP).

### Step 5: End of the study

- **Rec. 23.** Summary of results for laypersons:
  -
    - **Rec. 23.2**. Consider involving participants in the development and review of the summary.

### The gender perspective during the consent process for clinical studies

- **Rec. 25**. Adapt consent information by gender only when the strategy or study is directed at a single sex group.
- **Rec. 26.** In the case of women from different cultural backgrounds, consider using a cultural mediator with a gendered approach in order to bridge communication gaps.
- **Rec. 27.** Connect with the participant:
  -
    - **Rec. 27.1**. In research of a more sensitive nature (e.g. trials of vaccines against sexually transmitted diseases), it may be beneficial if the investigator in contact with the potential participant is of the same sex
    - **Rec. 27.2.** The major focus should be on connecting with the individual participant, rather than making gender-based assumptions

### The ICP in clinical studies involving minors

- **Rec. 29.** Information for children:
  - **Rec. 29.1.** Choose the information for the child on the basis of the minor’s level of maturity and their capacity of comprehension, not only on their age.
  - **Rec. 29.5.** Assess the minor’s capacity and understanding through:
    - Dialogue with the investigator (using a teach-back method).
    - Multiple choice questionnaires and/or open questions, such as MacCAT-CR test modified for children and adolescents.

All 53 recommendations were re-rated during the meeting. Results of second-round ratings are shown in Additional file 1.

After the second round, 42 recommendations were considered “appropriate” [12 of them were rated by all 14 panellists with scores between 7 and 9], 11 as “uncertain” (all of them with medians equals or above 6.5 but with disagreement), and none were considered “inappropriate”. Additional information in Additional file 1 lists the 53 recommendations and their results in the second round.

“General recommendations” and “Gender perspective during the consent process for clinical studies” were the two sections with a higher percentage of recommendations rated “uncertain”. Outside of these two sections, recommendations on how to assess understanding of informed consent and assent were the most questioned by the panellists.

## Discussion

Fifty-three recommendations were extracted and evaluated by the experts in this study. Most of the recommendations were considered “appropriate” and only a few changes were suggested. The modification of “appropriateness” to a more restrictive level resulted in a greater number of recommendations with the result "uncertain". This was very positive as it permitted a more fruitful discussion during the virtual meeting. It should be noted that on the original scale of appropriateness levels, all the recommendations evaluated would have been rated as "appropriate” after the two rounds.

The outcome of the virtual meeting was a better understanding of the panellists’ point of view, leading to the modification of some recommendations towards a more consensual content and wording. It was also an opportunity to explain the i-CONSENT rationale behind each recommendation, to clarify any doubts, and to allow all the experts to share their opinion on each recommendation. This approach benefitted the second-round evaluation, which better reflects the experts’ opinion.

It is also important to mention that none of the recommendations evaluated had “inappropriate” as result or had significant disagreement among the panellists, in which case they would have been removed from the guidelines. Furthermore, since there is no indication that any of the recommendations with the result "uncertain" were harmful (all of them had medians in the "appropriate" range and were proposed as a result of the research conducted during the project), they have been maintained in the guidelines, albeit in most cases with modifications derived from this study (Additional file 2).

The composition of the panel, with overrepresentation of patient representatives and women, was especially suitable for the objectives of the study, as two of the main objectives of the guidelines aim to put study participants in the centre of the process and to include a gender perspective. It is also important to note that most of the patient representatives were also investigators. Furthermore, due to COVID-19 pandemic, the in-person meeting was conducted remotely. The impact of these factors on the final results was thought to be low.

The following discussion focuses mainly on recommendations with the result “uncertain” after the first round of scoring.

### IC as a continuous communication process

The recommendation rated highest in the overall survey was to consider informed consent as a “two-way continuous communication process that begins at first contact with the potential participant and continues until the end of the study”.

**Fig. 3:**
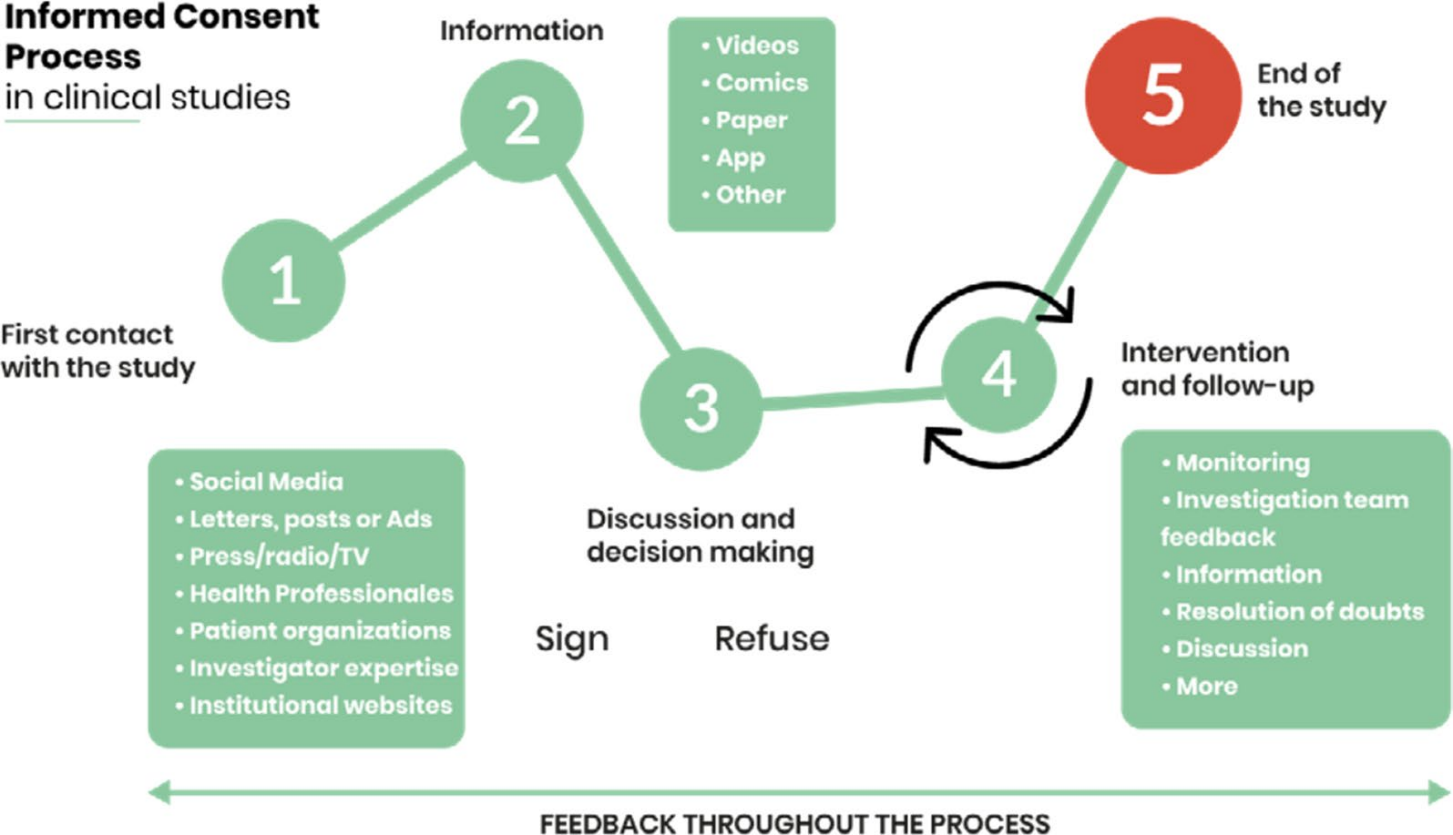
The informed consent process in clinical studies. *Source:* Guidelines for Tailoring the Informed Consent Process in Clinical Studies (7) (2021)

The ICP described in the i-CONSENT guidelines is a five-step process (Fig. 3). During this process, continuous feedback and communication between the potential or current participant and the research team is essential.

This “first contact”, as described in the i-CONSENT guidelines, aims to raise awareness of the study and provide essential study information before the recruitment process begins.

The panellists found it appropriate to consider the use of different channels to advertise the study, including social media and websites, in addition to the traditional routes. Even so, they highlighted the need to consider aspects of digital poverty and how the use of these channels actively excludes some pockets of society. The experts were also very cautious about recommending the use of email to reach out to potential participants. Recommendation 13 was reformulated after discussion to add some clarification and to remove any mention of decentralized clinical trials.

It is important to note that the Heads of Medicines Agencies (HMA) and the European Medicines Agency (EMA) [12] highlight the importance of understanding the implications of recruiting patients to research via social media.

Panellists agreed to recommend the inclusion of the following information during this first contact:

- The purpose of the research, the importance of the study, and expected duration;
- The target population with some inclusion/exclusion criteria (e.g., pregnant women between 18–40 years old);
- A brief description of the relevant study procedures (e.g., a routine blood sample); and
- The contact person at the study site.

A recommendation to provide the potential participant with all relevant information about the study (step 2) before the discussion with the investigator (step 3) was also considered appropriate, in order to ensure that they have had sufficient time to think about it and to prepare any questions.

The discussion between the potential participant and the investigator is clearly seen as a fundamental step of the ICP. The i-CONSENT project, however, strongly recommends separating both events (information and dialogue) during the process, because some potential participants make the decision to participate based solely on this interaction, without fully reading the patient information sheet (PIS) to the end. Traceability of dialogue is very difficult, and it is impossible to guarantee that all relevant information about the study has been delivered during the discussion.

Furthermore, it was considered appropriate that the participant be assured access to the information used during the ICP and knows how to access it throughout the study and for the period established by law.

The panellists also agreed on the importance of obtaining participants’ feedback on the ICP but they were unsure about how to obtain it (how often, when, how). The i-CONSENT project advocates obtaining feedback from participants to make the ICP more dynamic and responsive over time, adjusting it to the needs and preferences of the participants. The i-CONSENT also highlights the use of feedback tools, such as the Study Participant Feedback Questionnaire Toolkit [13] developed by Transcelerate Biopharma. The i-CONSENT similarly recommends obtaining feedback at different moments during the study: after signing the consent, during the intervention, and at the end of the study. Although none of the panellists advised against obtaining feedback at these three timepoints, and the majority of panellists were in favour of the concept, there was no agreement on the appropriateness of this recommendation, since some respondents considered that this could overburden investigators and/or participants.

### Health and digital literacy

Several studies identify health literacy as an important determinant of a patient’s capacity to provide fully informed consent [14, 15], and the i-CONSENT project sees the consent process as an opportunity to improve the health literacy of participants.

Panellists recognise the importance of health literacy but, in their opinion, it is not the duty of the research team to train participants in health and digital literacy or on how to detect fake news and unreliable sources. Some believed that these recommendations place an excessive burden on investigators.

Panellists believed that the emphasis should be on adapting the information to the target population’s preferences and needs, instead of adapting the population to the information. In this respect, one of the panellists stated: “I do not agree that the ICP should train participants in health literacy. The ambition for study teams MUST be to adjust information to the level(s) of participants, who might otherwise feel unsure or disrespected in their own right.”

Another idea highlighted by the panellists was the importance of creating information that does not require any further consultation, and that is easy for everyone to understand. In fact, there was no agreement on providing references or links to reliable sources of information.

Three recommendations considered appropriate by the panellists on this topic were to:

- Design the information to complete a possible lack of health literacy on the part of the potential participant.
- Use a glossary of terms to explain complex concepts.
- Use a layered approach for introducing study information, presenting the basic information in the general level and more specific messages in sub-layers. When using a document format (paper or pdf), these layers must be easily identifiable: the first layer will be in the main body and the sub-layers can come in a different format, such as in boxes or in different colours, or they can be presented in annexes.

### Co-creation as a key idea

Co-creation was highlighted as a key intervention to increase the quality and understanding of the ICP, including the development of consent information. All panellists believed it was appropriate to recommend the “use of co-design as a central concept. Include participants during the design and review of the study information. Pre-test the information with representatives of the target population”. This is in line with the findings of a previous study conducted with representatives of patient groups in the framework of the i-CONSENT project [16].

Co-creation is important when producing a PIS, and it is equally important to summarize results, decision aids or any material about health information, such as information leaflets, in plain language. This took on particular importance when recommendation 23.2 was discussed: this recommendation was considered too weak because the recommendation to involve participants in the development and review of the summary was to be "considered" rather than a "must". Therefore, the recommendation was reformulated as "Involve participants in the development and review of the summary of results".

The panellists believe that it is appropriate to recommend co-design as a central concept and to use quantitative and qualitative interdisciplinary methodologies to involve and obtain insights from the target population. This strategy reinforces the proposal made by Jackson et al. to use a participatory and mixed methods approach to design informed consent in a way that best suits the needs of participants [17].

### Tailoring the informed consent to potential participant preferences and needs

Usually during the ICP, patient information is only tailored to individual needs during in-person interactions. Normally, information materials are prepared without taking into account the preferences and needs of the target population.

Using a participatory and mixed method approach to develop informed consent will help identify the preferences and needs of the target population, including preferences regarding formats for presenting the information or the channels for contacting the research team.

Even so, as individual needs may differ from the general preferences of the target population, offering different possibilities during the ICP will help tailor it to the individual.

In this regard, most of the panellists felt that it was appropriate to offer potential participants a choice of more than one format for receiving information, and to provide different channels and formats for communicating with the research team.

As mentioned in the section on health and digital literacy, it is important to consider the benefit of presenting the information using a layered approach (especially if using a website). This approach will let the individual delve into the information they find most relevant or explore the explanations they need for better understanding.

### Use of technical and methodological innovations

The use of digital technologies during the ICP is increasing. Several studies have measured the impact of interventions using multimedia, audio–video, or gamification to provide information to patients or potential participants [18, 19].

Most panellists considered it appropriate to recommend the use of technical and methodological innovations during the ICP to facilitate the participant experience, including the use of new technologies and formats to deliver information (hyperlinked website, video, storytelling, comics, mobile applications). It is important to note that the adequacy of this approach should always be taken into account from a social, methodological, legal, and ethical point of view.

### Prepare inclusive information

According to the principle of justice and to ensure that the potential participant feels identified with the information provided, it is very important to prepare inclusive information and to implement an intercultural approach and a gender perspective.

The panellists believed that it is very appropriate to recommend procedures that incorporate a sensitive intercultural approach, empathizing with and being sensitive to the preferences and needs of people from different cultures, and adapting the consent process to their requirements as far as possible. Information should be provided in an easy-to-understand and culturally appropriate language and the participation of trained cross-cultural professionals in the study should be encouraged. It is also important to be aware that key concepts can be understood differently.

Literature on the gender perspective in the consent process is scant, and this is a controversial issue. Several studies exploring the ICP as a communication process have identified gender differences in this interaction. Even so, most authors agree that there are more common characteristics than differences, and that the differences identified are not categorical. Most studies that analysed differences in the understanding of informed consent in clinical trials by gender found no differences [4, 6, 20]. Indeed, the few studies that identified differences mostly found a better understanding by women [21–24]. Some studies also found that women were more inclined to read the entire PIS [25].

The panellists highlighted the importance of applying a gender perspective during the consent process, taking into account the influence of gender on health needs and concerns. This concern is in line with that expressed by the European Commission in the H2020 call “SwafS-17–2016—The ethics of informed consent in novel treatment including a gender perspective”.

The consent process must be conducted without reinforcing stereotypes. Using one PIS for men and another for women in the same study is difficult to justify and unacceptable in most cases. The best way to adapt informed consent to the target population is to take that population into account when designing the information via a process of co-creation. Moreover, in-person discussion will be essential to adapt the consent process to the particular characteristics of the potential participant, connecting with the individual without making gender-based assumptions.

Two actions that seemed to be beneficial in applying a gender perspective, but that failed to achieve agreement during the study, were:

- When the study is directed at a single sex group, it can be useful to take into account communication and eye-tracking differences when designing the materials.
- In research of a more sensitive nature (e.g., trials of vaccines against sexually transmitted diseases) it may be beneficial if the investigator in contact with the potential participant is of the same sex.

### Assessing understanding of information

Recommendations 18 and 29 highlighted the importance of the communication skills of the investigator. Panellists stated that the potential participant’s understanding has to be achieved through natural conversation. The experts were very critical of the use of questionnaires (especially with MacCAT) or the teach-back method. These, in their opinion, feel artificial and make the potential participant feel as if they are in an exam. However, given that the scientific literature has repeatedly pointed out the usefulness of techniques such as the teach-back method or questionnaires (including self-completion) to assess the level of understanding of potential participants [5, 19, 26, 27], we believe that while the best option is to have investigators with good communication skills that do not need to use these components, they can be a useful tool during verbal discussion at certain times.

Defining how to assess understanding is another important question that emerged during the discussion. The initial proposal for recommendation 18 was to verify that potential participants have understood “all” the information about the study, but this was considered unrealistic and unnecessary in most studies, and a more appropriate recommendation would be to verify that potential participants have understood “all relevant” information about the study. The information considered as relevant must be defined during the co-creation of the information, taking into account both perspectives (investigator/sponsor and potential participants).

In addition to providing clear and complete information, ensuring its understanding and replying to the doubts the potential participant, the panellists believe it is appropriate to recommend the use of decision-making tools to facilitate the process.

### Participant involvement at the end of the study

Participants should be informed at the end of the study about the results, and they should also be included in the early phases of disseminating the results.

A “thank you letter” is a good way of thanking the participant for their participation in the study and, if possible, giving a preview of the results and instructions on how to access the summary of results when it is ready.

Providing a summary of results is considered appropriate for all studies, not only clinical trials; participants should be involved in producing and reviewing the summary (as mentioned above). Other formats, including written reports, may be considered for the summary and the one that best suits the characteristics of the target population must be selected.

## Conclusion

The RAND/UCLA method has demonstrated validity for assessing the appropriateness of recommendations in ethical guidelines and can be used to obtain quantitative and qualitative information from panellists. Both rounds of rating provide very valuable information: the first round is very useful for detecting the recommendations for which there is already consensus regarding their status as “appropriate” or “inappropriate”. This made for a more productive meeting and focused the discussion on the recommendations rated as “uncertain” or without consensus. The changes between the first and second round are consistent with the initial ratings and the discussion. The inclusion of boxes in which to add comments on each recommendation during the rating rounds was very useful for a better understanding of the panellists' point of view and for making a qualitative interpretation of the results.

Most of the recommendations drawn from the i-CONSENT guidelines were considered “appropriate” by the panellists, and none was considered “inappropriate”. Only a few were rated as “uncertain” and this was always because of a lack of agreement. Medians for all recommendations fell between 6.5 and 9. Some “uncertain” recommendations have been reformulated or partially changed taking into account experts’ opinion (Additional file 2).

The four key aspects for improving the understanding of the ICP in clinical studies are:

- To consider consent a continuous two-way communication process that begins at the time of first contact with the potential participant, and continues until the end of the study;
- To improve investigators’ communication skills;
- To co-create information materials; and
- To use a layered approach, including information to compensate for a possible lack of health literacy on the part of the potential participant and a glossary of terms.

In addition to providing comprehensible information, it is essential to assess that all relevant information has been properly understood. It is recommended that understanding be assessed in a natural conversation and that the questions asked by the potential participant and their body language are evaluated by a well-trained researcher. This is preferable to the use of tools, such as the teach-back method or surveys, that can seem artificial and make people to feel as if they are in an exam.

## Supplementary Information

**Additional file 1.** List of recommendations rated by the panellists and results after round 2.

**Additional file 2.** Recommendations reformulated after 2 rounds of rating and virtual discussion.

## Data Availability

The anonymized data are available from the corresponding author upon request.

## Abbreviations

ICP: Informed consent process
PIS: Patient information sheet
PPRS: Personalised panellist rating sheet
Rec.: Recommendation

## Footnotes

1 Some panellists belonged to more than one group but were included in the one most representative for them.
