# Supplementary material for: Assessment of the appropriateness of the i-CONSENT guidelines recommendations for improving understanding of the informed consent process in clinical studies
Source: BMC Med Ethics. 2021 Oct 13;22:138. doi: 10.1186/s12910-021-00708-1 (PMC8513381; doi:10.1186/s12910-021-00708-1)
Supplement: Supplementary file 1 — Additional file 1. List of recommendations rated by the panellists and results after round 2. [file 12910_2021_708_MOESM1_ESM.docx]

**Additional file 1** List of recommendations rated by the panellists and results after round 2

| **General recommendations** | |
| --- | --- |
| **Recommendation** | **Result after the second round** |
| 1. Consent should be a two-way continuous communication process that begins at first contact with the potential participant, and continues until the end of the study. | Appropriate (Median: 9; Number of panellists rating outside the 7-9 point region: 0) |
| 2. Feedback from participants: |  |
| 2.1. Obtain feedback from participants about the consent process. | Appropriate (Median: 8,5; Number of panellists rating outside the 7-9 point region:1) |
| 2.2. Feedback should be obtained at all stages:   - About the experience before starting the study (to get during the first month of participation); - About the experience during the study (to get during the trial progress); - About the experience at the end of the study (to get during the last visit). | Uncertain (Median: 7; Number of panellists rating outside the 7-9 point region: 5) |
| 2.3. Do a de-briefing session with your team about the consent process using this information:   - Doing it after the study may help to improve the consent process of future studies; - Doing it during the study may also help to improve the process of the current study. | Appropriate (Median: 7,5; Number of panellists rating outside the 7-9 point region:3) |
| 3. The consent process should include technical and methodological innovations to facilitate the participant’s experience. Their appropriateness from a social, methodological, legal and ethical point of view should always be taken into consideration. | Appropriate (Median: 8; Number of panellists rating outside the 7-9 point region: 2) |
| 4. Digital and health literacy: |  |
| 4.1. Train your participants to improve their digital and health literacy. | Uncertain (Median: 7; Number of panellists rating outside the 7-9 point region: 6) |
| 4.2. Use a glossary of terms to explain complex concepts. | Appropriate (Median: 9; Number of panellists rating outside the 7-9 point region: 0) |
| 4.3. Use links to “further information”. | Uncertain (Median: 7; Number of panellists rating outside the 7-9 point region: 4) |
| 4.4. Provide participants with information on how to detect fake news and unreliable sources. | Uncertain (Median: 7; Number of panellists rating outside the 7-9 point region: 6) |
| **Recommendations for the elaboration of materials** | |
| **Recommendation** | **Result after the second round** |
| 5. Use interdisciplinary quantitative and qualitative methodologies to know your study population, interests and needs. It may be useful to:   - review the available literature on the target population (e.g. systematic or narrative literature review); - ask the target population directly (e.g. interviews; surveys; design thinking); - seek advice from experts (key informant interviews; brainstorming…); - observe the target population; - analyse their interactions on social media and blogs. | Appropriate (Median: 9; Number of panellists rating outside the 7-9 point region: 2) |
| 6. Use co-design as a central concept. Include the participants during the design and review of the study information materials. Pre-test the materials with representatives of the target population. | Appropriate (Median: 9; Number of panellists rating outside the 7-9 point region: 0) |
| 7. Use a layered approach for presenting study information, putting in the general level the basic information and in sub-layers the more specific one. When using a document format (paper or pdf), these layers must be easily identifiable, the first layer will be the main body and the sub-layers can be in a different format, such as in boxes or in different colours, or they can be in Annexes. | Appropriate (Median: 8,5; Number of panellists rating outside the 7-9 point region: 1) |
| 8. Give potential participants a choice of more than one format for receiving information. | Appropriate (Median: 8,5; Number of panellists rating outside the 7-9 point region: 2) |
| 9. Providing different channels and formats to communicate with the research team. | Appropriate (Median: 8; Number of panellists rating outside the 7-9 point region: 1) |
| 10. Design the information to complete a possible lack of health literacy of the potential participant. | Appropriate (Median: 8; Number of panellists rating outside the 7-9 point region: 0) |
| 11. Provided references to reliable sources of information. | Uncertain (Median: 7,5; Number of panellists rating outside the 7-9 point region: 4) |
| 12. If using placebo include a short description of the placebo effect (positive and negative). | Appropriate (Median: 9; Number of panellists rating outside the 7-9 point region: 2) |
| **Step 1: First contact with the potential participant** | |
| **Recommendation** | **Result after the second round** |
| 13. Due to its growing use among the population and the appearance of Decentralised Clinical Trials, consider: |  |
| 13.1. Different channels to advertise the study: | Appropriate (Median: 8; Number of panellists rating outside the 7-9 point region: 3) |
| - 13.1.1. Use social media. | Appropriate (Median: 7; Number of panellists rating outside the 7-9 point region: 3) |
| - 13.1.2. Information on websites. | Appropriate (Median: 8,5; Number of panellists rating outside the 7-9 point region: 3) |
| - 13.1.3. Use email. | Uncertain (Median: 7; Number of panellists rating outside the 7-9 point region: 6) |
| 14. The information provided in the first contact with the potential participant should include: |  |
| 14.1. The purpose of the research, the importance of the study and expected duration. | Appropriate (Median: 9; Number of panellists rating outside the 7-9 point region: 0) |
| 14.2. The target population with some inclusion/exclusion criteria (e.g. pregnant women between 18-40 years old). | Appropriate (Median: 9; Number of panellists rating outside the 7-9 point region: 1) |
| 14.3. A brief description of the relevant study procedures (e.g. a routine blood sample). | Appropriate (Median: 9; Number of panellists rating outside the 7-9 point region: 0) |
| 14.4. Contact person at the study site. | Appropriate (Median: 9; Number of panellists rating outside the 7-9 point region: 0) |
| **Step 2: Provision of information** | |
| **Recommendation** | **Result after the second round** |
| 15. Provide the participant with all relevant information about the study before the discussion with the investigator, ensuring that they have sufficient time to consider it and to prepare any questions that they may have. | Appropriate (Median: 7,5; Number of panellists rating outside the 7-9 point region: 3) |
| 16. Consider new technologies and formats to deliver information (website with hyperlinks, video, storytelling, comics, mobile Apps). | Appropriate (Median: 8; Number of panellists rating outside the 7-9 point region: 2) |
| **Step 3: Discussion and Decision Making** | |
| **Recommendation** | **Result after the second round** |
| 17. Strengthen the communication skills for the investigators. Investigators should receive appropriate training to ensure that verbal communication is delivered in a balanced and complete manner. | Appropriate (Median: 9; Number of panellists rating outside the 7-9 point region: 0) |
| 18. Check that potential participants have understood all the study information by:   - Interview: Teach-back or teach-to-goal methods can be helpful. - Questionnaires: such as the Quality of Informed Consent (QuIC); Deaconess Informed Consent comprehension test (DICCT); or the Brief Informed Consent Evaluation Protocol (BICEP) " | Uncertain (Median: 8; Number of panellists rating outside the 7-9 point region: 5) |
| 19. Use decision aids to facilitate the decision-making process. | Appropriate (Median: 8; Number of panellists rating outside the 7-9 point region: 3) |
| **Step 4: Intervention and Follow-up** | |
| **Recommendation** | **Result after the second round** |
| 20. Ensure that participant has access to the information used during the recruitment process and knows how to access to it (the information must be accessible during all the study and until the term established by law). | Appropriate (Median: 8,5; Number of panellists rating outside the 7-9 point region: 1) |
| **Step 5: Completion of the study** | |
| **Recommendation** | **Result after the second round** |
| 21. Thank participants for taking part in the study using a “thank you letter”. | Appropriate (Median: 9; Number of panellists rating outside the 7-9 point region: 2) |
| 22. Include participants in the first steps of result dissemination. | Appropriate (Median: 8,5; Number of panellists rating outside the 7-9 point region: 2) |
| 23. Summary of results: |  |
| 23.1. Do a lay language summary of results. | Appropriate (Median: 9; Number of panellists rating outside the 7-9 point region: 0) |
| 23.2. Consider involving participants in the development and review of the summary. | Appropriate (Median: 8; Number of panellists rating outside the 7-9 point region: 3) |
| 23.3. Consider other formats, as well as written, for providing a summary and choose one that best suits the characteristics of the target population. | Appropriate (Median: 8; Number of panellists rating outside the 7-9 point region: 1) |
| **The gender perspective during the consent process for clinical studies** | |
| **Recommendation** | **Result after the second round** |
| 24. Inclusive approach: |  |
| 24.1. Ensure that materials are inclusive and do not reinforce gender stereotypes. | Appropriate (Median: 9; Number of panellists rating outside the 7-9 point region: 0) |
| 24.2. Take into account the ways in which gender influences health needs and concerns. | Appropriate (Median: 9; Number of panellists rating outside the 7-9 point region: 0) |
| 24.3. Pre-test and re-test messages, concepts, and intended program formats with women and men to ensure that they work well for both. | Appropriate (Median: 8,5; Number of panellists rating outside the 7-9 point region: 1) |
| 25. Adapt consent materials by gender only when the strategy or study is directed to a single sex group. | Uncertain (Median: 7; Number of panellists rating outside the 7-9 point region: 4; “Do not know”: 2) |
| 26. In the case of women coming from different cultural backgrounds, consider using a cultural mediator with a gendered approach in order to bridge communication gaps. | Uncertain (Median: 8; Number of panellists rating outside the 7-9 point region: 3; “Do not know”: 1) |
| 27. Connect with the participant: |  |
| 27.1. In research of a more sensitive nature (e.g. trials of vaccines against sexually transmitted diseases) it may be beneficial if the investigator in contact with the potential participant is of his/her same sex. | Uncertain (Median: 7; Number of panellists rating outside the 7-9 point region: 5; “Do not know”: 1) |
| 27.2. The major focus should be on connecting with the individual participant, rather than making gender-based assumptions. | Appropriate (Median: 8; Number of panellists rating outside the 7-9 point region: 2) |
| **The informed consent process in clinical studies involving minors** | |
| **Recommendation** | **Result after the second round** |
| 28. Involve minors in the decision-making process in accordance to their age and maturity level. | Appropriate (Median: 9; Number of panellists rating outside the 7-9 point region:1; “Do not know”: 1 ) |
| 29. Information materials for children: |  |
| 29.1. Choose the information materials for the child basing your decision on the minor’s level of maturity and his/her capacity of comprehension, not only on his/her age. | Appropriate (Median: 8; Number of panellists rating outside the 7-9 point region: 2; “Do not know”: 1) |
| 29.2. Provide information in a format (not just the language) in accordance to the age of the children. | Appropriate (Median: 9; Number of panellists rating outside the 7-9 point region: 1; “Do not know”: 1) |
| 29.3. Use simple graphics or pictures to accompany information. | Appropriate (Median: 9; Number of panellists rating outside the 7-9 point region: 0; “Do not know”: 1) |
| 29.4. Use interactive approaches (video, storytelling, gamification tools, website with hyperlinks, mobile Apps for 12+ years old). | Appropriate (Median: 8; Number of panellists rating outside the 7-9 point region: 2; “Do not know”: 1) |
| 29.5. Assess the minor’s capacity and understanding through:   - Dialogue with the investigator (using a tech-back method) - Multiple choices questionnaires and /or open questions - MacCAT-CR test modified for children and adolescents | Uncertain (Median: 6,5; Number of panellists rating outside the 7-9 point region: 7) |
| **The informed consent process in clinical studies involving people from different cultural and religious backgrounds** | |
| **Recommendation** | **Result after the second round** |
| 30. Intercultural approach: |  |
| 30.1. Adopt procedures that incorporate an intercultural sensitive approach. | Appropriate (Median: 9; Number of panellists rating outside the 7-9 point region: 1; “Do not know”: 1) |
| 30.2. Be aware that key concepts can be understood differently. | Appropriate (Median: 9; Number of panellists rating outside the 7-9 point region: 0) |
| 30.3. Empathize/Sensibilize. | Appropriate (Median: 9; Number of panellists rating outside the 7-9 point region: 1; “Do not know”: 1) |
| 30.4. When possible, adapt the consent process:   - Provide information in an easy-to-understand and culturally appropriate language; - Promote the participation of trained cross-cultural professionals in the study | Appropriate (Median: 8,5; Number of panellists rating outside the 7-9 point region: 2) |
