# Supplementary material for: Assessment of the appropriateness of the i-CONSENT guidelines recommendations for improving understanding of the informed consent process in clinical studies
Source: BMC Med Ethics. 2021 Oct 13;22:138. doi: 10.1186/s12910-021-00708-1 (PMC8513381; doi:10.1186/s12910-021-00708-1)
Supplement: Supplementary file 2 — Additional file 2. Recommendations reformulated after 2 rounds of rating and virtual discussion. [file 12910_2021_708_MOESM2_ESM.docx]

**Additional file 2** Recommendations reformulated after 2 rounds of rating and virtual discussion

| **Original** | **Final** |
| --- | --- |
| 2.2. Feedback should be obtained at all stages:   - About the experience before starting the study (to get during the first month of participation); - About the experience during the study (to get during the trial progress); - About the experience at the end of the study (to get during the last visit). | 2.2. Feedback should be obtained at all stages   - About the experience before starting the study (to get during the first month of participation); - About the experience during the study (to get during the trial progress); - About the experience at the end of the study (to get during the last visit).   Feedback should be obtained in a way that avoids overloading investigators and/or participants |
| 4. Digital and health literacy: | 4. Digital and health literacy:  Study information should be adapted to the health literacy level of the potential participant to enable them to make an appropriate decision about whether or not to take part. ICP also provides a unique opportunity to improve potential participant’s health and digital literacy. |
| 11. Provided references to reliable sources of information. | 11. The materials must be easy to understand and as complete as possible, so that it is not necessary to consult external information. Even so, it is recommended to provide references to reliable sources of information for people who want to dig deeper. |
| 13. Due to its growing use among the population and the appearance of Decentralised Clinical Trials, consider: | 13. Due to the growing use of social media and Information and Communication Technologies among the population, consider: |
| 18. Check that potential participants have understood all the study information by:   - Interview: Teach-back or teach-to-goal methods can be helpful. - Questionnaires: such as the Quality of Informed Consent (QuIC); Deaconess Informed Consent comprehension test (DICCT); or the Brief Informed Consent Evaluation Protocol (BICEP) " | 18. Verify that potential participants have understood all relevant information about the study through a conversation with the investigator. If the researcher does not have adequate communication skills, it is recommended that he/she reinforces them.  Even so, there are some tools or methods that can be used to assess comprehension, although they are not as recommended as natural conversation, because they can seem artificial and make the potential participant feel evaluated or on an exam. Among these tools or methods we find:   - Interview: Teach-back or teach-to-goal methods. - Questionnaires: such as the Quality of Informed Consent (QuIC); Deaconess Informed Consent comprehension test (DICCT); or the Brief Informed Consent Evaluation Protocol (BICEP) |
| 23.2. Consider involving participants in the development and review of the summary. | 23.2. Involve participants in the development and review of the summary of results |
| 29.5. Assess the minor’s capacity and understanding through:   - Dialogue with the investigator (using a tech-back method) - Multiple choices questionnaires and /or open questions - MacCAT-CR test modified for children and adolescents | 29.5 Assess the minor’s capacity and understanding through a conversation with the investigator. If the researcher does not have adequate communication skills, it is recommended that he/she reinforce them.  Even so, there are some methods that can be used, although they are not as recommended as natural conversation, because they can seem artificial and make the minor feel evaluated or on an exam. Among these methods we find:   - Dialogue with the investigator (using a tech-back method) - Multiple choices questionnaires and /or open questions |
